# Crystal structure of poly[[[μ_4_-5-(9*H*-carbazol-9-yl)isophthalato][μ_3_-5-(9*H*-carbazol-9-yl)isophthalato]bis­(di­methyl­formamide)(methanol)dizinc] di­methyl­formamide monosolvate]

**DOI:** 10.1107/S2056989015013961

**Published:** 2015-07-31

**Authors:** Liubov M. Lifshits, Charles Campana, Jeremy K. Klosterman

**Affiliations:** a Department of Chemistry and Center for Photochemical Sciences, Bowling Green State University, Bowling Green, OH 43403, USA; b Bruker AXS Inc., Madison, Wisconsin, USA

**Keywords:** crystal structure, zinc, metal–organic framework, laminar solids

## Abstract

The structure of the polymeric title compound, {[Zn_2_(C_20_H_11_NO_4_)_2_(C_3_H_7_NO)_2_(CH_3_OH)]·C_3_H_7_NO}_*n*_, comprises carbazolylisophthalate moieties connecting dimetallic tetra­carboxyl­ate zinc secondary building units (SBUs) parallel to [100] and [010], leading to a layer-like arrangement parallel to (001). Each SBU consists of two Zn atoms in slightly distorted tetra­hedral and octa­hedral coordination environments [Zn⋯Zn = 3.5953 (6) Å]. Three carboxyl­ate groups bridge the two Zn atoms in a μ_2_-*O*:*O*′ mode, whereas the fourth coordinates through a single carboxyl­ate O atom (μ_1_-*O*). The O atoms of two di­methyl­formamide (DMF) and one methanol mol­ecule complete the Zn coordination spheres. The methanol ligand inter­acts with the noncoordinating DMF mol­ecule *via* an O—H⋯O hydrogen bond of medium strength. Carbazoles between the layers inter­digitate through weak C—H⋯.π inter­actions to form a laminar solid stacked along [010]. Two kinds of C—H⋯π inter­actions are present, both with a distance of 2.64 Å, between the H atoms and the centroids, and a third C—H⋯π inter­action, where the aromatic H atom is located above the carbazole N-atom lone pair (H⋯N = 2.89 Å). Several C—H⋯O inter­actions occur between the coordinating DMF mol­ecule, the DMF solvent mol­ecule, and ligating carboxyl­ate O atoms.

## Related literature   

For solid-state emission behavior and inter­molecular packing inter­actions for a closely related compound where methanol is replaced by ethanol, see: Lifshits *et al.* (2015 ▸). This compound and the title compound are solvatomorphs with identical space groups and comparable lattice parameters. Except for the identity of the coordinating solvent, *viz.* MeOH *versus* EtOH, the other structural components are the same.
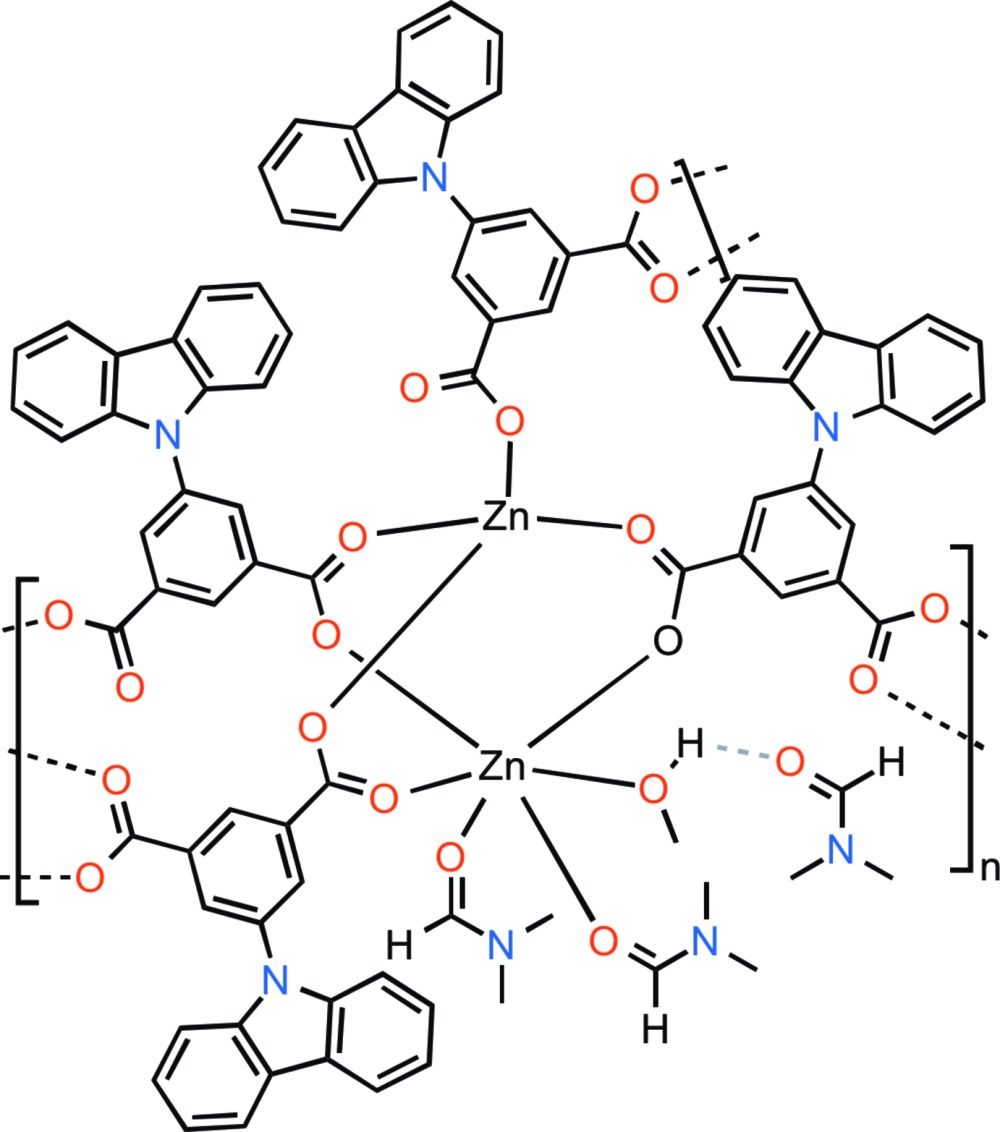


## Experimental   

### Crystal data  

[Zn_2_(C_20_H_11_NO_4_)_2_(C_3_H_7_NO)_2_(CH_3_OH)]·C_3_H_7_NO
*M* *_r_* = 1040.66
Orthorhombic, 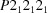

*a* = 10.2867 (4) Å
*b* = 17.0328 (7) Å
*c* = 26.8808 (11) Å
*V* = 4709.8 (3) Å^3^
*Z* = 4
Cu *K*α radiation
μ = 1.84 mm^−1^
*T* = 100 K
0.40 × 0.22 × 0.22 mm

### Data collection  

Bruker D8 Venture CMOS diffractometer
Absorption correction: multi-scan (*SADABS*; Bruker, 2015 ▸) *T*_min_ = 0.53, *T*_max_ = 0.68
41928 measured reflections
8611 independent reflections
8561 reflections with *I* > 2σ(*I*)
*R*_int_ = 0.019

### Refinement  

*R*[*F* ^2^ > 2σ(*F* ^2^)] = 0.019
*wR*(*F* ^2^) = 0.045
*S* = 1.08
8611 reflections
629 parameters
546 restraints
H-atom parameters constrained
Δρ_max_ = 0.41 e Å^−3^
Δρ_min_ = −0.18 e Å^−3^
Absolute structure: Flack *x* determined using 3716 quotients [(*I* ^+^) − (*I* ^−^)]/[(*I* ^+^) + (*I* ^−^)] (Parsons *et al*, 2013 ▸)
Absolute structure parameter: 0.005 (3)

### 

Data collection: *APEX3* (Bruker, 2015 ▸); cell refinement: *SAINT* (Bruker, 2015 ▸); data reduction: *SAINT*; program(s) used to solve structure: *SHELXS97* (Sheldrick, 2008 ▸); program(s) used to refine structure: *SHELXL2014* (Sheldrick, 2015 ▸); molecular graphics: *ShelXle* (Hübschle *et al.*, 2011 ▸); software used to prepare material for publication: *APEX3*.

## Supplementary Material

Crystal structure: contains datablock(s) global, I. DOI: 10.1107/S2056989015013961/wm5180sup1.cif

Structure factors: contains datablock(s) I. DOI: 10.1107/S2056989015013961/wm5180Isup2.hkl

**Fig. 1.**
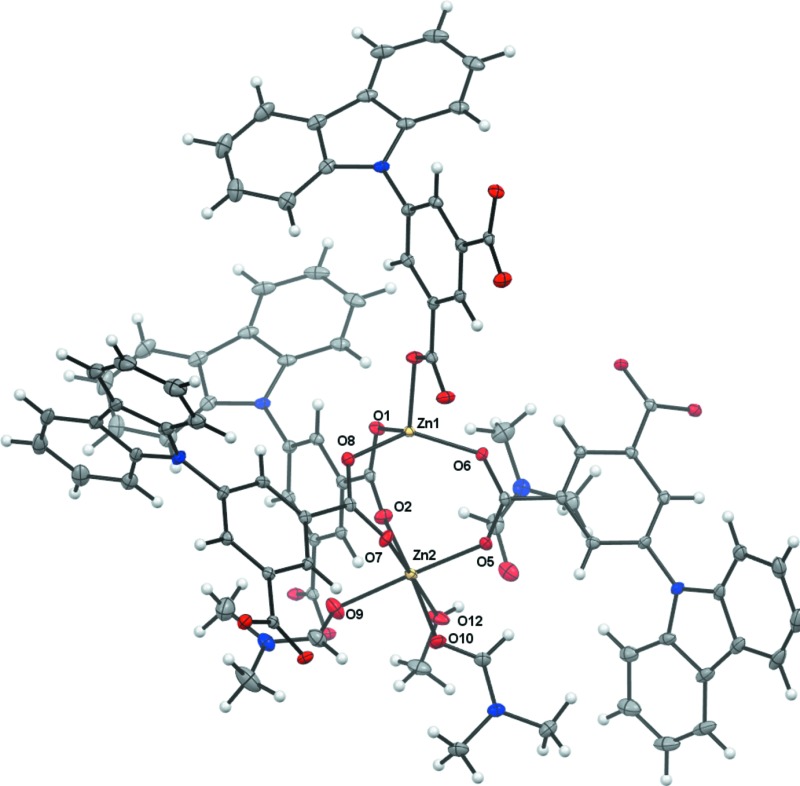
Part of the title structure showing the dimetallic Zn secondary building unit with displacement ellipsoids drawn at the 50% probability level.

. DOI: 10.1107/S2056989015013961/wm5180fig1.tif
Part of the title structure showing the dimetallic Zn secondary building unit with displacement ellipsoids drawn at the 50% probability level.

**Fig. 2.**
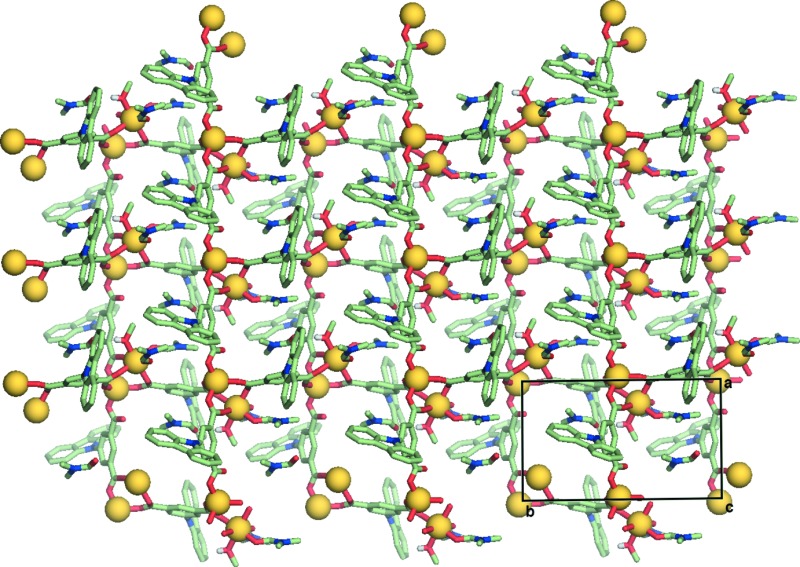
Crystal structure of the title complex in a projection down [001]. Colour code: light green: C, dark blue: N, red: O, white: H, yellow spheres: Zn.

. DOI: 10.1107/S2056989015013961/wm5180fig2.tif
Crystal structure of the title complex in a projection down [001]. Colour code: light green: C, dark blue: N, red: O, white: H, yellow spheres: Zn.

**Fig. 3.**
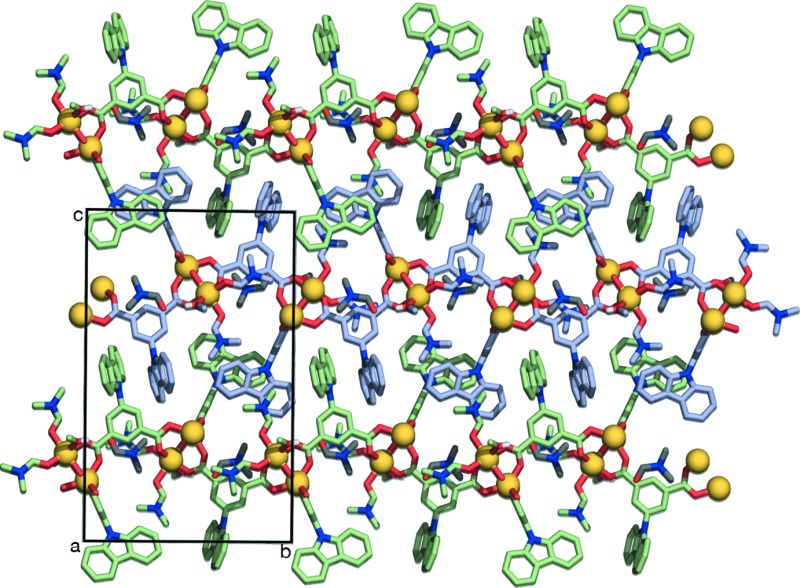
Crystal structure of the title complex in a projection down [100] showing the layer-like arrangement of the Zn secondary building units and the carbazole moieties parallel to (001), and the interdigitation of carbazoles to form stacked layers along [010]. Colour code as in Fig. 2.

. DOI: 10.1107/S2056989015013961/wm5180fig3.tif
Crystal structure of the title complex in a projection down [100] showing the layer-like arrangement of the Zn secondary building units and the carbazole moieties parallel to (001), and the inter­digitation of carbazoles to form stacked layers along [010]. Colour code as in Fig. 2.

**Fig. 4.**
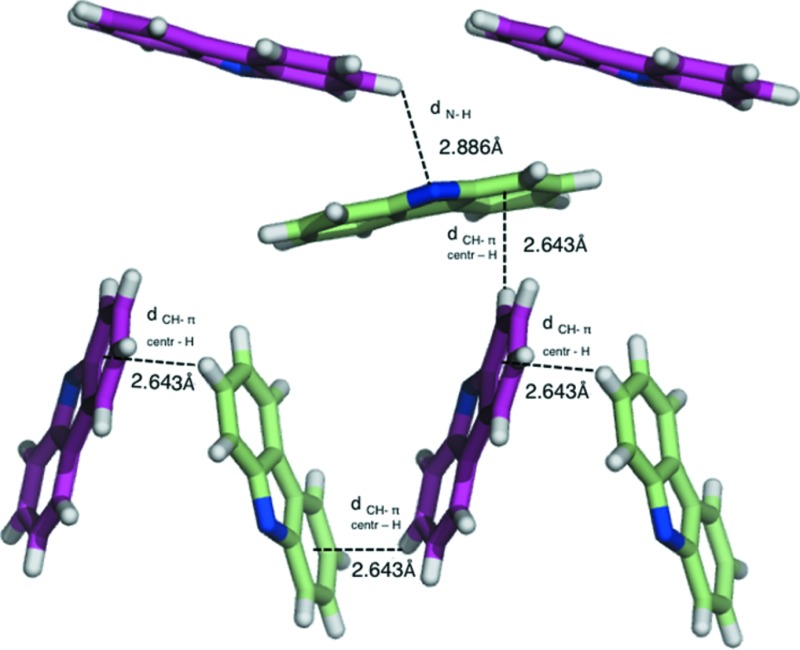
C—H···π interactions between carbazole moieties in the title structure.

. DOI: 10.1107/S2056989015013961/wm5180fig4.tif
C—H⋯π inter­actions between carbazole moieties in the title structure.

**Fig. 5.**
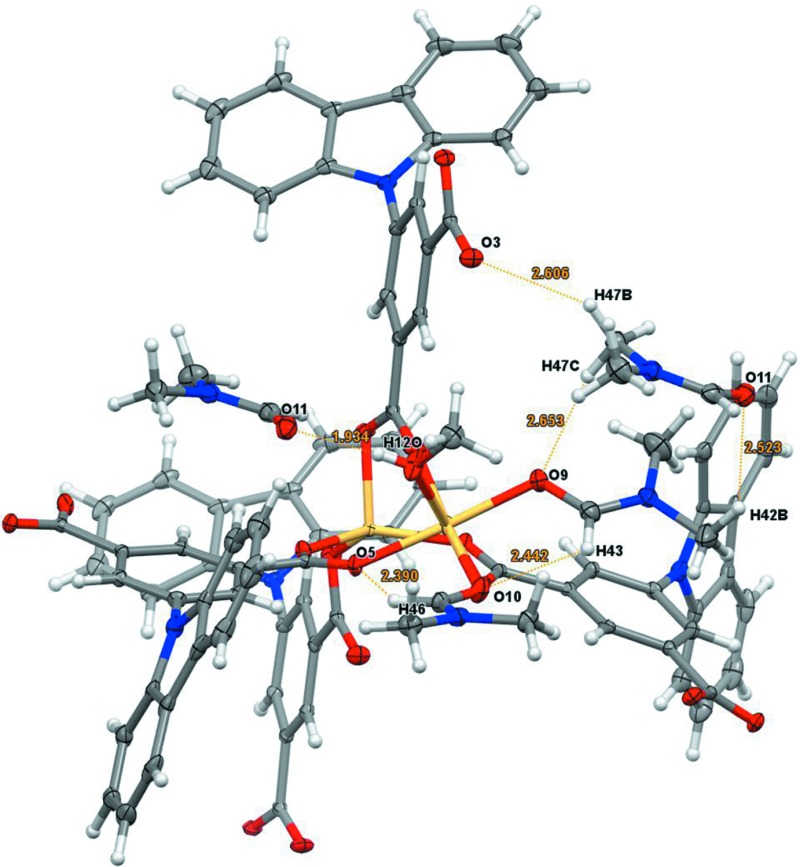
C—H···O interactions between the coordinating DMF molecule, the DMF solvate molecule, and ligating carboxylate oxygen atoms.

. DOI: 10.1107/S2056989015013961/wm5180fig5.tif
C—H⋯O inter­actions between the coordinating DMF mol­ecule, the DMF solvate mol­ecule, and ligating carboxyl­ate oxygen atoms.

CCDC reference: 1408495

Additional supporting information:  crystallographic information; 3D view; checkCIF report

## Figures and Tables

**Table 1:** Hydrogen-bond geometry (, ) *Cg*1, *Cg*3, *Cg*4 and *Cg*6 are the centroids of the N1/C1/C6/C7/C12, C1C6, C7C12 and C21C26 rings, respectively.

*D*H*A*	*D*H	H*A*	*D* *A*	*D*H*A*
O12H12*O*O11	0.76	1.93	2.696(2)	174
C42H42*B*O11^i^	0.98	2.52	3.327(3)	139
C43H43O10	0.95	2.44	3.028(3)	120
C46H46O5	0.95	2.39	2.995(2)	121
C47H47*B*O3^ii^	0.98	2.61	3.417(3)	140
C47H47*C*O9^iii^	0.98	2.65	3.608(3)	165
C2H2*Cg*6^iv^	0.95	2.64	3.517(2)	153
C10H10*Cg*3^v^	0.95	2.64	3.418(2)	139
C44H44*B* *Cg*4^vi^	0.98	2.91	3.596(2)	128
C45H45*B* *Cg*1^vi^	0.98	2.85	3.411(2)	117

Symmetry codes: (i) 
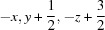
; (ii) 
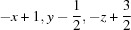
; (iii) 
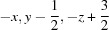
; (iv) 
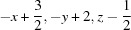
; (v) 
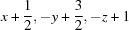
; (vi) 
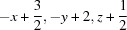
.

## supplementary crystallographic information

### S1. Synthesis and crystallization

The title compound was prepared in an analogous manner to Zn Cbz - EtOH (Lifshits *et al.*, 2015). Single crystals were obtained by the solvothermal reaction of 5-(9*H*-carbazol-9-yl)-isophthalic acid and zinc nitrate in DMF/MeOH at room temperature overnight.

### S2. Refinement

All H-atom positions were located from difference maps, then refined with isotropic temperature factors. Subsequently, positional restraints were added for all hydrogen atoms and *U*_iso_ values for hydrogen atoms were restrained to 1.2*U*_eq_ of the attached atom.

### Crystal data

**Table:**

[Zn_2_(C_20_H_11_NO_4_)_2_(C_3_H_7_NO)_2_(CH_3_OH)]·C_3_H_7_NO	*D*_x_ = 1.468 Mg m^−^^3^
*M**_r_* = 1040.66	Cu *K*α radiation, λ = 1.54178 Å
Orthorhombic, *P*2_1_2_1_2_1_	Cell parameters from 9734 reflections
*a* = 10.2867 (4) Å	θ = 5.4–68.3°
*b* = 17.0328 (7) Å	µ = 1.84 mm^−^^1^
*c* = 26.8808 (11) Å	*T* = 100 K
*V* = 4709.8 (3) Å^3^	Prism, clear colourless
*Z* = 4	0.40 × 0.22 × 0.22 mm
*F*(000) = 2152	

### Data collection

**Table:**

Bruker D8 Venture CMOS diffractometer	8611 independent reflections
Radiation source: ImuS micro-focus source with QUAZAR optics	8561 reflections with *I* > 2σ(*I*)
Mirrors monochromator	*R*_int_ = 0.019
ω– and φ–scans	θ_max_ = 68.2°, θ_min_ = 3.1°
Absorption correction: multi-scan (*SADABS*; Bruker, 2015)	*h* = −12→11
*T*_min_ = 0.53, *T*_max_ = 0.68	*k* = −20→20
41928 measured reflections	*l* = −31→32

### Refinement

**Table:**

Refinement on *F*^2^	Hydrogen site location: mixed
Least-squares matrix: full	H-atom parameters constrained
*R*[*F*^2^ > 2σ(*F*^2^)] = 0.019	*w* = 1/[σ^2^(*F*_o_^2^) + (0.0221*P*)^2^ + 1.2023*P*] where *P* = (*F*_o_^2^ + 2*F*_c_^2^)/3
*wR*(*F*^2^) = 0.045	(Δ/σ)_max_ = 0.004
*S* = 1.08	Δρ_max_ = 0.41 e Å^−^^3^
8611 reflections	Δρ_min_ = −0.18 e Å^−^^3^
629 parameters	Absolute structure: Flack *x* determined using 3716 quotients [(I+)-(I-)]/[(I+)+(I-)] (Parsons *et al*, 2013)
546 restraints	Absolute structure parameter: 0.005 (3)
0 constraints	

### Special details

**Table:**

Geometry. All e.s.d.'s (except the e.s.d. in the dihedral angle between two l.s. planes) are estimated using the full covariance matrix. The cell e.s.d.'s are taken into account individually in the estimation of e.s.d.'s in distances, angles and torsion angles; correlations between e.s.d.'s in cell parameters are only used when they are defined by crystal symmetry. An approximate (isotropic) treatment of cell e.s.d.'s is used for estimating e.s.d.'s involving l.s. planes.

### Fractional atomic coordinates and isotropic or equivalent isotropic displacement parameters (Å^2^)

**Table:**

	*x*	*y*	*z*	*U*_iso_*/*U*_eq_	
Zn1	0.52406 (2)	0.98187 (2)	0.67608 (2)	0.00831 (6)	
Zn2	0.30186 (2)	1.08249 (2)	0.75728 (2)	0.01117 (6)	
N1	1.01341 (16)	0.86300 (9)	0.51641 (6)	0.0129 (3)	
C1	0.95145 (19)	0.89610 (12)	0.47539 (7)	0.0146 (4)	
C2	0.9057 (2)	0.97254 (13)	0.46907 (8)	0.0180 (4)	
H2	0.9108	1.0102	0.4951	0.022*	
C3	0.8523 (2)	0.99110 (14)	0.42298 (8)	0.0248 (5)	
H3	0.8192	1.0425	0.4176	0.03*	
C4	0.8461 (2)	0.93588 (15)	0.38421 (8)	0.0277 (5)	
H4	0.8103	0.9506	0.353	0.033*	
C5	0.8915 (2)	0.86053 (15)	0.39113 (8)	0.0247 (5)	
H5	0.8872	0.8233	0.3648	0.03*	
C6	0.9441 (2)	0.83924 (13)	0.43731 (8)	0.0177 (4)	
C7	0.99990 (19)	0.76781 (12)	0.45673 (8)	0.0171 (4)	
C8	1.0156 (2)	0.69190 (13)	0.43782 (8)	0.0221 (4)	
H8	0.9852	0.679	0.4055	0.027*	
C9	1.0761 (2)	0.63599 (13)	0.46707 (9)	0.0238 (5)	
H9	1.0863	0.5841	0.4547	0.029*	
C10	1.1228 (2)	0.65440 (13)	0.51475 (9)	0.0223 (5)	
H10	1.1652	0.6151	0.5339	0.027*	
C11	1.1076 (2)	0.72951 (13)	0.53442 (8)	0.0178 (4)	
H11	1.1396	0.7423	0.5666	0.021*	
C12	1.04404 (19)	0.78507 (12)	0.50537 (7)	0.0150 (4)	
C13	1.0189 (2)	0.89772 (10)	0.56479 (7)	0.0119 (4)	
C14	1.13827 (19)	0.91247 (11)	0.58751 (7)	0.0119 (4)	
H14	1.2173	0.8991	0.5713	0.014*	
C15	1.13966 (19)	0.94734 (11)	0.63455 (7)	0.0115 (4)	
C16	1.0238 (2)	0.96674 (10)	0.65848 (7)	0.0113 (3)	
H16	1.0258	0.9911	0.6903	0.014*	
C17	0.90537 (19)	0.95048 (11)	0.63589 (7)	0.0107 (4)	
C18	0.90292 (19)	0.91604 (11)	0.58874 (7)	0.0117 (4)	
H18	0.8222	0.9052	0.573	0.014*	
C19	1.26733 (18)	0.96743 (10)	0.65857 (7)	0.0112 (4)	
O1	1.36949 (13)	0.93922 (8)	0.63956 (5)	0.0135 (3)	
O2	1.26507 (14)	1.01114 (9)	0.69596 (5)	0.0166 (3)	
C20	0.77903 (18)	0.97111 (11)	0.66134 (7)	0.0118 (4)	
O3	0.77960 (14)	1.01396 (9)	0.69850 (5)	0.0174 (3)	
O4	0.67727 (13)	0.94091 (8)	0.64176 (5)	0.0145 (3)	
N2	0.39399 (18)	0.83315 (10)	0.95005 (6)	0.0147 (3)	
C21	0.4863 (2)	0.83442 (11)	0.98791 (7)	0.0155 (4)	
C22	0.6179 (2)	0.81631 (12)	0.98552 (8)	0.0188 (4)	
H22	0.6558	0.7959	0.956	0.023*	
C23	0.6920 (3)	0.82912 (12)	1.02792 (8)	0.0245 (5)	
H23	0.7825	0.8181	1.0272	0.029*	
C24	0.6360 (3)	0.85803 (13)	1.07181 (8)	0.0270 (5)	
H24	0.689	0.8669	1.1002	0.032*	
C25	0.5048 (3)	0.87372 (12)	1.07420 (8)	0.0264 (5)	
H25	0.4673	0.8919	1.1044	0.032*	
C26	0.4265 (2)	0.86281 (12)	1.03187 (8)	0.0190 (4)	
C27	0.2928 (2)	0.87905 (11)	1.01955 (8)	0.0197 (4)	
C28	0.1854 (3)	0.90598 (12)	1.04676 (8)	0.0265 (5)	
H28	0.1933	0.917	1.0813	0.032*	
C29	0.0688 (3)	0.91627 (14)	1.02287 (10)	0.0302 (5)	
H29	−0.0041	0.9346	1.0411	0.036*	
C30	0.0554 (2)	0.90011 (13)	0.97173 (10)	0.0281 (5)	
H30	−0.0264	0.9081	0.9561	0.034*	
C31	0.1596 (2)	0.87272 (12)	0.94368 (8)	0.0212 (4)	
H31	0.1509	0.8617	0.9092	0.025*	
C32	0.2771 (2)	0.86227 (11)	0.96842 (8)	0.0167 (4)	
C33	0.4234 (2)	0.81833 (11)	0.89901 (7)	0.0124 (4)	
C34	0.42478 (19)	0.88080 (11)	0.86558 (7)	0.0121 (4)	
H34	0.4032	0.9322	0.8765	0.015*	
C35	0.45792 (18)	0.86756 (11)	0.81612 (7)	0.0109 (4)	
C36	0.48732 (19)	0.79181 (11)	0.79992 (7)	0.0106 (4)	
H36	0.5115	0.7829	0.7663	0.013*	
C37	0.48112 (19)	0.72937 (10)	0.83313 (7)	0.0107 (3)	
C38	0.45010 (18)	0.74252 (11)	0.88311 (7)	0.0118 (4)	
H38	0.4474	0.7	0.9059	0.014*	
C39	0.45579 (18)	0.93481 (11)	0.77969 (7)	0.0106 (4)	
O5	0.39906 (14)	0.99586 (8)	0.79345 (5)	0.0146 (3)	
O6	0.51058 (13)	0.92271 (7)	0.73846 (5)	0.0134 (3)	
C40	0.50276 (18)	0.64683 (11)	0.81456 (7)	0.0107 (4)	
O7	0.53555 (15)	0.63806 (8)	0.77011 (5)	0.0153 (3)	
O8	0.48208 (14)	0.59232 (7)	0.84533 (5)	0.0131 (3)	
N3	0.18493 (18)	1.29637 (10)	0.69906 (7)	0.0203 (4)	
C41	0.1585 (3)	1.28253 (18)	0.64698 (9)	0.0359 (6)	
H41A	0.1585	1.2259	0.6405	0.043*	
H41B	0.2258	1.3078	0.6267	0.043*	
H41C	0.0733	1.3045	0.6384	0.043*	
C42	0.1993 (3)	1.37942 (14)	0.71429 (12)	0.0368 (6)	
H42A	0.2282	1.3818	0.749	0.044*	
H42B	0.1156	1.4063	0.711	0.044*	
H42C	0.2638	1.4051	0.693	0.044*	
C43	0.2047 (2)	1.23963 (13)	0.73133 (9)	0.0231 (4)	
H43	0.2304	1.2537	0.7641	0.028*	
O9	0.19190 (17)	1.16868 (9)	0.72161 (6)	0.0249 (3)	
N4	0.23401 (18)	1.14908 (10)	0.90142 (6)	0.0165 (4)	
C44	0.1897 (2)	1.23038 (12)	0.90000 (8)	0.0212 (4)	
H44A	0.2421	1.2599	0.876	0.025*	
H44B	0.1991	1.2539	0.9331	0.025*	
H44C	0.0982	1.2319	0.89	0.025*	
C45	0.2081 (2)	1.10314 (13)	0.94622 (7)	0.0206 (4)	
H45A	0.1143	1.0943	0.9494	0.025*	
H45B	0.2396	1.1318	0.9754	0.025*	
H45C	0.2529	1.0525	0.9439	0.025*	
C46	0.2883 (2)	1.11725 (12)	0.86199 (7)	0.0170 (4)	
H46	0.317	1.0644	0.8644	0.02*	
O10	0.30448 (16)	1.15212 (8)	0.82162 (5)	0.0193 (3)	
N5	0.18348 (18)	0.76557 (10)	0.75996 (7)	0.0198 (4)	
C47	0.1482 (2)	0.70971 (13)	0.79858 (9)	0.0228 (5)	
H47A	0.1474	0.7363	0.8309	0.027*	
H47B	0.2117	0.6668	0.7992	0.027*	
H47C	0.0616	0.6884	0.7916	0.027*	
C48	0.2438 (3)	0.73480 (16)	0.71458 (9)	0.0299 (5)	
H48A	0.2654	0.7784	0.6923	0.036*	
H48B	0.183	0.6992	0.6978	0.036*	
H48C	0.3232	0.7062	0.7232	0.036*	
C49	0.1679 (2)	0.84238 (13)	0.76636 (8)	0.0218 (4)	
H49	0.1952	0.876	0.7402	0.026*	
O11	0.12041 (16)	0.87346 (9)	0.80389 (6)	0.0238 (3)	
C50	0.0105 (2)	1.06679 (13)	0.80194 (9)	0.0265 (5)	
H50A	0.0221	1.1239	0.8027	0.032*	
H50B	−0.0201	1.0487	0.8345	0.032*	
H50C	−0.0536	1.053	0.7764	0.032*	
O12	0.13112 (15)	1.03020 (9)	0.79064 (6)	0.0238 (3)	
H12O	0.1229	0.9862	0.7954	0.029*	

### Atomic displacement parameters (Å^2^)

**Table:**

	*U*^11^	*U*^22^	*U*^33^	*U*^12^	*U*^13^	*U*^23^
Zn1	0.00790 (11)	0.00827 (11)	0.00877 (11)	0.00017 (9)	0.00090 (9)	0.00012 (9)
Zn2	0.01339 (12)	0.00866 (11)	0.01146 (12)	0.00126 (9)	0.00188 (9)	−0.00029 (9)
N1	0.0120 (8)	0.0150 (7)	0.0118 (7)	0.0003 (6)	0.0004 (6)	−0.0050 (6)
C1	0.0096 (9)	0.0205 (10)	0.0136 (9)	−0.0038 (7)	0.0005 (7)	−0.0014 (7)
C2	0.0167 (10)	0.0191 (10)	0.0184 (10)	−0.0045 (8)	−0.0014 (8)	−0.0004 (8)
C3	0.0262 (12)	0.0244 (11)	0.0238 (11)	−0.0048 (9)	−0.0047 (9)	0.0069 (9)
C4	0.0296 (13)	0.0364 (13)	0.0172 (11)	−0.0084 (10)	−0.0071 (9)	0.0048 (9)
C5	0.0254 (12)	0.0343 (12)	0.0143 (10)	−0.0077 (10)	−0.0024 (9)	−0.0064 (9)
C6	0.0140 (10)	0.0240 (10)	0.0149 (9)	−0.0051 (8)	0.0017 (7)	−0.0052 (8)
C7	0.0112 (10)	0.0222 (10)	0.0178 (9)	−0.0035 (8)	0.0034 (7)	−0.0074 (8)
C8	0.0193 (11)	0.0234 (10)	0.0236 (10)	−0.0057 (9)	0.0057 (9)	−0.0122 (8)
C9	0.0197 (11)	0.0186 (10)	0.0331 (12)	−0.0035 (9)	0.0119 (9)	−0.0110 (9)
C10	0.0139 (10)	0.0188 (10)	0.0342 (12)	0.0015 (8)	0.0079 (9)	−0.0025 (9)
C11	0.0120 (9)	0.0200 (10)	0.0214 (10)	0.0001 (8)	0.0025 (8)	−0.0047 (8)
C12	0.0103 (9)	0.0174 (9)	0.0174 (9)	−0.0020 (7)	0.0042 (7)	−0.0055 (7)
C13	0.0128 (9)	0.0111 (8)	0.0119 (8)	−0.0007 (7)	−0.0006 (7)	−0.0014 (7)
C14	0.0100 (9)	0.0117 (9)	0.0140 (9)	0.0002 (7)	0.0009 (7)	−0.0005 (7)
C15	0.0113 (9)	0.0091 (8)	0.0141 (9)	−0.0005 (7)	0.0000 (7)	0.0017 (7)
C16	0.0138 (9)	0.0095 (8)	0.0106 (8)	−0.0002 (7)	0.0010 (7)	0.0008 (6)
C17	0.0112 (9)	0.0090 (8)	0.0118 (9)	−0.0002 (7)	0.0016 (7)	0.0017 (7)
C18	0.0092 (9)	0.0107 (8)	0.0152 (9)	−0.0007 (7)	−0.0014 (7)	0.0009 (7)
C19	0.0106 (9)	0.0104 (9)	0.0127 (9)	−0.0005 (7)	0.0001 (7)	0.0032 (7)
O1	0.0084 (7)	0.0156 (7)	0.0166 (7)	0.0000 (5)	−0.0013 (5)	−0.0023 (5)
O2	0.0141 (7)	0.0206 (7)	0.0151 (7)	−0.0022 (6)	−0.0027 (5)	−0.0063 (6)
C20	0.0109 (9)	0.0104 (8)	0.0140 (9)	0.0015 (7)	0.0021 (7)	0.0032 (7)
O3	0.0146 (7)	0.0222 (7)	0.0155 (7)	0.0014 (6)	0.0021 (5)	−0.0068 (6)
O4	0.0099 (7)	0.0173 (7)	0.0163 (7)	0.0003 (5)	0.0016 (5)	−0.0037 (5)
N2	0.0219 (9)	0.0129 (8)	0.0092 (8)	0.0017 (7)	0.0028 (7)	−0.0004 (6)
C21	0.0284 (11)	0.0080 (8)	0.0100 (8)	−0.0010 (8)	−0.0010 (8)	0.0009 (7)
C22	0.0277 (12)	0.0129 (9)	0.0157 (10)	0.0024 (8)	−0.0009 (8)	0.0016 (8)
C23	0.0364 (13)	0.0148 (9)	0.0221 (11)	0.0026 (10)	−0.0090 (10)	0.0031 (8)
C24	0.0468 (15)	0.0184 (10)	0.0159 (10)	−0.0035 (10)	−0.0105 (10)	0.0022 (8)
C25	0.0545 (17)	0.0136 (9)	0.0110 (9)	−0.0080 (10)	0.0022 (10)	−0.0012 (7)
C26	0.0357 (12)	0.0091 (9)	0.0122 (9)	−0.0044 (8)	0.0049 (8)	0.0001 (7)
C27	0.0348 (12)	0.0099 (9)	0.0145 (9)	−0.0059 (9)	0.0092 (9)	−0.0029 (7)
C28	0.0403 (14)	0.0167 (10)	0.0226 (11)	−0.0058 (10)	0.0180 (10)	−0.0065 (8)
C29	0.0340 (13)	0.0191 (10)	0.0376 (13)	−0.0027 (10)	0.0224 (11)	−0.0056 (10)
C30	0.0239 (12)	0.0215 (11)	0.0388 (13)	0.0007 (9)	0.0104 (10)	−0.0011 (10)
C31	0.0243 (11)	0.0159 (10)	0.0233 (11)	−0.0007 (8)	0.0063 (9)	−0.0007 (8)
C32	0.0251 (11)	0.0085 (8)	0.0165 (10)	−0.0006 (8)	0.0090 (8)	−0.0009 (7)
C33	0.0147 (9)	0.0145 (9)	0.0079 (8)	0.0004 (8)	0.0004 (7)	−0.0019 (7)
C34	0.0130 (9)	0.0104 (9)	0.0131 (9)	0.0005 (7)	0.0009 (7)	−0.0013 (7)
C35	0.0099 (9)	0.0112 (8)	0.0117 (9)	0.0000 (7)	−0.0011 (7)	0.0009 (7)
C36	0.0095 (9)	0.0133 (8)	0.0091 (8)	0.0007 (7)	−0.0007 (7)	−0.0009 (7)
C37	0.0088 (8)	0.0113 (8)	0.0119 (8)	0.0009 (7)	−0.0009 (7)	−0.0007 (7)
C38	0.0117 (9)	0.0117 (9)	0.0119 (9)	−0.0007 (7)	0.0007 (7)	0.0022 (7)
C39	0.0098 (9)	0.0110 (9)	0.0110 (8)	−0.0005 (7)	−0.0022 (7)	0.0007 (7)
O5	0.0215 (7)	0.0096 (6)	0.0127 (6)	0.0037 (5)	0.0014 (5)	0.0006 (5)
O6	0.0155 (7)	0.0140 (6)	0.0106 (6)	0.0024 (5)	0.0033 (5)	0.0030 (5)
C40	0.0085 (9)	0.0117 (8)	0.0120 (9)	0.0009 (7)	−0.0018 (7)	−0.0006 (7)
O7	0.0211 (7)	0.0141 (6)	0.0107 (6)	0.0067 (6)	0.0010 (6)	−0.0020 (5)
O8	0.0160 (7)	0.0097 (6)	0.0136 (6)	0.0005 (5)	0.0011 (5)	−0.0010 (5)
N3	0.0154 (9)	0.0186 (8)	0.0270 (9)	0.0020 (7)	−0.0017 (7)	0.0010 (7)
C41	0.0376 (15)	0.0446 (15)	0.0256 (12)	0.0053 (12)	−0.0028 (10)	0.0056 (11)
C42	0.0266 (13)	0.0203 (11)	0.0634 (18)	0.0022 (10)	−0.0086 (13)	−0.0019 (11)
C43	0.0193 (10)	0.0224 (10)	0.0276 (11)	0.0038 (9)	−0.0044 (9)	−0.0020 (9)
O9	0.0241 (8)	0.0159 (7)	0.0349 (8)	0.0039 (6)	−0.0082 (7)	0.0007 (6)
N4	0.0170 (9)	0.0172 (8)	0.0152 (8)	−0.0004 (7)	0.0013 (7)	−0.0041 (7)
C44	0.0235 (11)	0.0173 (10)	0.0229 (10)	0.0020 (9)	0.0041 (9)	−0.0065 (8)
C45	0.0227 (11)	0.0245 (10)	0.0146 (10)	−0.0006 (9)	0.0028 (8)	−0.0007 (8)
C46	0.0201 (10)	0.0151 (9)	0.0156 (10)	0.0021 (8)	0.0006 (8)	−0.0024 (7)
O10	0.0295 (8)	0.0143 (6)	0.0142 (6)	0.0018 (6)	0.0048 (7)	−0.0035 (5)
N5	0.0182 (9)	0.0209 (8)	0.0202 (8)	−0.0020 (7)	−0.0010 (7)	0.0009 (7)
C47	0.0216 (11)	0.0207 (10)	0.0260 (11)	−0.0009 (9)	−0.0006 (9)	0.0025 (9)
C48	0.0301 (13)	0.0351 (13)	0.0247 (12)	−0.0039 (11)	0.0027 (10)	−0.0041 (10)
C49	0.0163 (10)	0.0234 (10)	0.0257 (11)	−0.0037 (8)	−0.0019 (8)	0.0057 (9)
O11	0.0189 (8)	0.0186 (7)	0.0339 (9)	−0.0006 (6)	0.0013 (7)	0.0009 (6)
C50	0.0168 (11)	0.0255 (11)	0.0374 (12)	0.0029 (9)	0.0072 (9)	−0.0048 (9)
O12	0.0192 (8)	0.0142 (7)	0.0381 (9)	0.0015 (6)	0.0150 (7)	0.0019 (6)

### Geometric parameters (Å, º)

**Table:**

Zn1—O4	1.9548 (14)	C27—C28	1.402 (3)
Zn1—O6	1.9614 (13)	C27—C32	1.413 (3)
Zn1—O8^i^	1.9683 (13)	C28—C29	1.372 (4)
Zn1—O1^ii^	2.0047 (14)	C28—H28	0.95
Zn2—O5	2.0303 (14)	C29—C30	1.409 (4)
Zn2—O7^i^	2.0580 (14)	C29—H29	0.95
Zn2—O2^ii^	2.0826 (14)	C30—C31	1.391 (3)
Zn2—O9	2.0867 (15)	C30—H30	0.95
Zn2—O10	2.0974 (14)	C31—C32	1.391 (3)
Zn2—O12	2.1638 (15)	C31—H31	0.95
N1—C1	1.393 (3)	C33—C38	1.388 (3)
N1—C12	1.396 (3)	C33—C34	1.393 (3)
N1—C13	1.430 (2)	C34—C35	1.391 (3)
C1—C2	1.395 (3)	C34—H34	0.95
C1—C6	1.411 (3)	C35—C36	1.395 (3)
C2—C3	1.392 (3)	C35—C39	1.507 (3)
C2—H2	0.95	C36—C37	1.390 (3)
C3—C4	1.405 (3)	C36—H36	0.95
C3—H3	0.95	C37—C38	1.399 (3)
C4—C5	1.378 (4)	C37—C40	1.508 (2)
C4—H4	0.95	C38—H38	0.95
C5—C6	1.402 (3)	C39—O5	1.248 (2)
C5—H5	0.95	C39—O6	1.260 (2)
C6—C7	1.443 (3)	C40—O7	1.250 (2)
C7—C8	1.399 (3)	C40—O8	1.262 (2)
C7—C12	1.415 (3)	O7—Zn2^iv^	2.0580 (14)
C8—C9	1.383 (4)	O8—Zn1^iv^	1.9683 (13)
C8—H8	0.95	N3—C43	1.315 (3)
C9—C10	1.404 (3)	N3—C41	1.445 (3)
C9—H9	0.95	N3—C42	1.480 (3)
C10—C11	1.393 (3)	C41—H41A	0.98
C10—H10	0.95	C41—H41B	0.98
C11—C12	1.390 (3)	C41—H41C	0.98
C11—H11	0.95	C42—H42A	0.98
C13—C18	1.391 (3)	C42—H42B	0.98
C13—C14	1.395 (3)	C42—H42C	0.98
C14—C15	1.397 (3)	C43—O9	1.243 (3)
C14—H14	0.95	C43—H43	0.95
C15—C16	1.395 (3)	N4—C46	1.315 (3)
C15—C19	1.503 (3)	N4—C44	1.458 (3)
C16—C17	1.389 (3)	N4—C45	1.461 (3)
C16—H16	0.95	C44—H44A	0.98
C17—C18	1.397 (3)	C44—H44B	0.98
C17—C20	1.510 (3)	C44—H44C	0.98
C18—H18	0.95	C45—H45A	0.98
C19—O2	1.251 (2)	C45—H45B	0.98
C19—O1	1.263 (2)	C45—H45C	0.98
O1—Zn1^iii^	2.0047 (14)	C46—O10	1.248 (3)
O2—Zn2^iii^	2.0825 (14)	C46—H46	0.95
C20—O3	1.237 (2)	N5—C49	1.329 (3)
C20—O4	1.279 (2)	N5—C47	1.454 (3)
N2—C32	1.391 (3)	N5—C48	1.465 (3)
N2—C21	1.392 (3)	C47—H47A	0.98
N2—C33	1.427 (2)	C47—H47B	0.98
C21—C22	1.390 (3)	C47—H47C	0.98
C21—C26	1.417 (3)	C48—H48A	0.98
C22—C23	1.388 (3)	C48—H48B	0.98
C22—H22	0.95	C48—H48C	0.98
C23—C24	1.402 (4)	C49—O11	1.240 (3)
C23—H23	0.95	C49—H49	0.95
C24—C25	1.377 (4)	C50—O12	1.421 (3)
C24—H24	0.95	C50—H50A	0.98
C25—C26	1.406 (3)	C50—H50B	0.98
C25—H25	0.95	C50—H50C	0.98
C26—C27	1.442 (4)	O12—H12O	0.7642
			
O4—Zn1—O6	106.09 (6)	C32—C27—C26	107.09 (19)
O4—Zn1—O8^i^	103.24 (6)	C29—C28—C27	119.1 (2)
O6—Zn1—O8^i^	137.62 (5)	C29—C28—H28	120.4
O4—Zn1—O1^ii^	106.21 (5)	C27—C28—H28	120.4
O6—Zn1—O1^ii^	100.16 (6)	C28—C29—C30	121.2 (2)
O8^i^—Zn1—O1^ii^	100.24 (6)	C28—C29—H29	119.4
O5—Zn2—O7^i^	96.05 (6)	C30—C29—H29	119.4
O5—Zn2—O2^ii^	92.55 (6)	C31—C30—C29	121.3 (2)
O7^i^—Zn2—O2^ii^	97.64 (5)	C31—C30—H30	119.4
O5—Zn2—O9	176.68 (7)	C29—C30—H30	119.4
O7^i^—Zn2—O9	87.28 (6)	C30—C31—C32	116.9 (2)
O2^ii^—Zn2—O9	87.04 (6)	C30—C31—H31	121.5
O5—Zn2—O10	90.56 (6)	C32—C31—H31	121.5
O7^i^—Zn2—O10	91.40 (6)	C31—C32—N2	128.81 (18)
O2^ii^—Zn2—O10	170.07 (6)	C31—C32—C27	122.6 (2)
O9—Zn2—O10	89.32 (6)	N2—C32—C27	108.6 (2)
O5—Zn2—O12	84.38 (6)	C38—C33—C34	120.68 (17)
O7^i^—Zn2—O12	175.73 (6)	C38—C33—N2	120.16 (17)
O2^ii^—Zn2—O12	86.58 (6)	C34—C33—N2	119.16 (17)
O9—Zn2—O12	92.30 (7)	C35—C34—C33	119.69 (17)
O10—Zn2—O12	84.34 (6)	C35—C34—H34	120.2
C1—N1—C12	108.65 (16)	C33—C34—H34	120.2
C1—N1—C13	124.80 (16)	C34—C35—C36	120.11 (17)
C12—N1—C13	125.29 (16)	C34—C35—C39	119.62 (17)
N1—C1—C2	128.95 (18)	C36—C35—C39	120.21 (16)
N1—C1—C6	108.73 (18)	C37—C36—C35	119.81 (16)
C2—C1—C6	122.29 (19)	C37—C36—H36	120.1
C3—C2—C1	117.0 (2)	C35—C36—H36	120.1
C3—C2—H2	121.5	C36—C37—C38	120.32 (16)
C1—C2—H2	121.5	C36—C37—C40	119.59 (16)
C2—C3—C4	121.7 (2)	C38—C37—C40	120.05 (16)
C2—C3—H3	119.1	C33—C38—C37	119.33 (17)
C4—C3—H3	119.1	C33—C38—H38	120.3
C5—C4—C3	120.5 (2)	C37—C38—H38	120.3
C5—C4—H4	119.7	O5—C39—O6	127.29 (17)
C3—C4—H4	119.7	O5—C39—C35	116.57 (16)
C4—C5—C6	119.4 (2)	O6—C39—C35	116.14 (16)
C4—C5—H5	120.3	C39—O5—Zn2	133.92 (12)
C6—C5—H5	120.3	C39—O6—Zn1	134.38 (12)
C5—C6—C1	119.1 (2)	O7—C40—O8	125.74 (17)
C5—C6—C7	133.8 (2)	O7—C40—C37	117.86 (16)
C1—C6—C7	107.15 (18)	O8—C40—C37	116.36 (15)
C8—C7—C12	119.4 (2)	C40—O7—Zn2^iv^	128.02 (12)
C8—C7—C6	133.9 (2)	C40—O8—Zn1^iv^	121.14 (12)
C12—C7—C6	106.67 (17)	C43—N3—C41	123.3 (2)
C9—C8—C7	118.8 (2)	C43—N3—C42	120.3 (2)
C9—C8—H8	120.6	C41—N3—C42	116.3 (2)
C7—C8—H8	120.6	N3—C41—H41A	109.5
C8—C9—C10	121.3 (2)	N3—C41—H41B	109.5
C8—C9—H9	119.4	H41A—C41—H41B	109.5
C10—C9—H9	119.4	N3—C41—H41C	109.5
C11—C10—C9	120.9 (2)	H41A—C41—H41C	109.5
C11—C10—H10	119.6	H41B—C41—H41C	109.5
C9—C10—H10	119.6	N3—C42—H42A	109.5
C12—C11—C10	117.7 (2)	N3—C42—H42B	109.5
C12—C11—H11	121.2	H42A—C42—H42B	109.5
C10—C11—H11	121.2	N3—C42—H42C	109.5
C11—C12—N1	129.34 (18)	H42A—C42—H42C	109.5
C11—C12—C7	121.91 (19)	H42B—C42—H42C	109.5
N1—C12—C7	108.75 (18)	O9—C43—N3	124.0 (2)
C18—C13—C14	120.81 (16)	O9—C43—H43	118.0
C18—C13—N1	118.69 (17)	N3—C43—H43	118.0
C14—C13—N1	120.50 (18)	C43—O9—Zn2	121.99 (15)
C13—C14—C15	118.82 (18)	C46—N4—C44	120.20 (18)
C13—C14—H14	120.6	C46—N4—C45	121.41 (17)
C15—C14—H14	120.6	C44—N4—C45	118.25 (17)
C16—C15—C14	120.63 (18)	N4—C44—H44A	109.5
C16—C15—C19	119.67 (17)	N4—C44—H44B	109.5
C14—C15—C19	119.64 (17)	H44A—C44—H44B	109.5
C17—C16—C15	120.06 (17)	N4—C44—H44C	109.5
C17—C16—H16	120.0	H44A—C44—H44C	109.5
C15—C16—H16	120.0	H44B—C44—H44C	109.5
C16—C17—C18	119.76 (18)	N4—C45—H45A	109.5
C16—C17—C20	120.68 (16)	N4—C45—H45B	109.5
C18—C17—C20	119.55 (17)	H45A—C45—H45B	109.5
C13—C18—C17	119.91 (17)	N4—C45—H45C	109.5
C13—C18—H18	120.0	H45A—C45—H45C	109.5
C17—C18—H18	120.0	H45B—C45—H45C	109.5
O2—C19—O1	124.52 (17)	O10—C46—N4	124.13 (19)
O2—C19—C15	117.67 (17)	O10—C46—H46	117.9
O1—C19—C15	117.81 (16)	N4—C46—H46	117.9
C19—O1—Zn1^iii^	108.90 (12)	C46—O10—Zn2	116.49 (12)
C19—O2—Zn2^iii^	168.46 (13)	C49—N5—C47	121.42 (19)
O3—C20—O4	124.97 (17)	C49—N5—C48	120.73 (19)
O3—C20—C17	119.93 (17)	C47—N5—C48	117.77 (18)
O4—C20—C17	115.10 (16)	N5—C47—H47A	109.5
C20—O4—Zn1	108.79 (12)	N5—C47—H47B	109.5
C32—N2—C21	108.95 (16)	H47A—C47—H47B	109.5
C32—N2—C33	125.96 (17)	N5—C47—H47C	109.5
C21—N2—C33	124.11 (18)	H47A—C47—H47C	109.5
C22—C21—N2	128.86 (18)	H47B—C47—H47C	109.5
C22—C21—C26	122.47 (19)	N5—C48—H48A	109.5
N2—C21—C26	108.58 (19)	N5—C48—H48B	109.5
C23—C22—C21	117.5 (2)	H48A—C48—H48B	109.5
C23—C22—H22	121.2	N5—C48—H48C	109.5
C21—C22—H22	121.2	H48A—C48—H48C	109.5
C22—C23—C24	121.3 (2)	H48B—C48—H48C	109.5
C22—C23—H23	119.3	O11—C49—N5	125.0 (2)
C24—C23—H23	119.3	O11—C49—H49	117.5
C25—C24—C23	120.7 (2)	N5—C49—H49	117.5
C25—C24—H24	119.7	O12—C50—H50A	109.5
C23—C24—H24	119.7	O12—C50—H50B	109.5
C24—C25—C26	119.8 (2)	H50A—C50—H50B	109.5
C24—C25—H25	120.1	O12—C50—H50C	109.5
C26—C25—H25	120.1	H50A—C50—H50C	109.5
C25—C26—C21	118.1 (2)	H50B—C50—H50C	109.5
C25—C26—C27	135.0 (2)	C50—O12—Zn2	128.06 (13)
C21—C26—C27	106.75 (18)	C50—O12—H12O	107.3
C28—C27—C32	118.9 (2)	Zn2—O12—H12O	124.2
C28—C27—C26	134.0 (2)		
			
C12—N1—C1—C2	−178.7 (2)	C26—C21—C22—C23	−1.8 (3)
C13—N1—C1—C2	13.6 (3)	C21—C22—C23—C24	1.0 (3)
C12—N1—C1—C6	−1.0 (2)	C22—C23—C24—C25	0.8 (3)
C13—N1—C1—C6	−168.66 (18)	C23—C24—C25—C26	−1.8 (3)
N1—C1—C2—C3	177.2 (2)	C24—C25—C26—C21	1.0 (3)
C6—C1—C2—C3	−0.4 (3)	C24—C25—C26—C27	−173.6 (2)
C1—C2—C3—C4	−0.8 (3)	C22—C21—C26—C25	0.8 (3)
C2—C3—C4—C5	1.0 (4)	N2—C21—C26—C25	−175.98 (17)
C3—C4—C5—C6	0.0 (4)	C22—C21—C26—C27	176.83 (18)
C4—C5—C6—C1	−1.2 (3)	N2—C21—C26—C27	0.0 (2)
C4—C5—C6—C7	−179.4 (2)	C25—C26—C27—C28	−6.7 (4)
N1—C1—C6—C5	−176.59 (19)	C21—C26—C27—C28	178.2 (2)
C2—C1—C6—C5	1.4 (3)	C25—C26—C27—C32	173.3 (2)
N1—C1—C6—C7	2.1 (2)	C21—C26—C27—C32	−1.7 (2)
C2—C1—C6—C7	−179.99 (18)	C32—C27—C28—C29	−0.9 (3)
C5—C6—C7—C8	−4.4 (4)	C26—C27—C28—C29	179.2 (2)
C1—C6—C7—C8	177.2 (2)	C27—C28—C29—C30	0.1 (3)
C5—C6—C7—C12	176.0 (2)	C28—C29—C30—C31	0.4 (4)
C1—C6—C7—C12	−2.4 (2)	C29—C30—C31—C32	−0.1 (3)
C12—C7—C8—C9	−1.1 (3)	C30—C31—C32—N2	177.3 (2)
C6—C7—C8—C9	179.4 (2)	C30—C31—C32—C27	−0.7 (3)
C7—C8—C9—C10	−0.7 (3)	C21—N2—C32—C31	179.0 (2)
C8—C9—C10—C11	1.0 (3)	C33—N2—C32—C31	10.0 (3)
C9—C10—C11—C12	0.5 (3)	C21—N2—C32—C27	−2.8 (2)
C10—C11—C12—N1	178.3 (2)	C33—N2—C32—C27	−171.78 (18)
C10—C11—C12—C7	−2.3 (3)	C28—C27—C32—C31	1.2 (3)
C1—N1—C12—C11	178.9 (2)	C26—C27—C32—C31	−178.88 (19)
C13—N1—C12—C11	−13.4 (3)	C28—C27—C32—N2	−177.18 (18)
C1—N1—C12—C7	−0.6 (2)	C26—C27—C32—N2	2.8 (2)
C13—N1—C12—C7	167.07 (18)	C32—N2—C33—C38	−117.0 (2)
C8—C7—C12—C11	2.6 (3)	C21—N2—C33—C38	75.7 (3)
C6—C7—C12—C11	−177.73 (19)	C32—N2—C33—C34	63.0 (3)
C8—C7—C12—N1	−177.81 (18)	C21—N2—C33—C34	−104.4 (2)
C6—C7—C12—N1	1.8 (2)	C38—C33—C34—C35	−2.4 (3)
C1—N1—C13—C18	57.1 (3)	N2—C33—C34—C35	177.66 (18)
C12—N1—C13—C18	−108.6 (2)	C33—C34—C35—C36	1.1 (3)
C1—N1—C13—C14	−123.2 (2)	C33—C34—C35—C39	178.31 (17)
C12—N1—C13—C14	71.1 (3)	C34—C35—C36—C37	1.2 (3)
C18—C13—C14—C15	−1.1 (3)	C39—C35—C36—C37	−175.98 (17)
N1—C13—C14—C15	179.23 (17)	C35—C36—C37—C38	−2.3 (3)
C13—C14—C15—C16	0.3 (3)	C35—C36—C37—C40	175.25 (17)
C13—C14—C15—C19	−176.94 (17)	C34—C33—C38—C37	1.2 (3)
C14—C15—C16—C17	0.9 (3)	N2—C33—C38—C37	−178.79 (18)
C19—C15—C16—C17	178.11 (17)	C36—C37—C38—C33	1.1 (3)
C15—C16—C17—C18	−1.2 (3)	C40—C37—C38—C33	−176.43 (17)
C15—C16—C17—C20	179.98 (17)	C34—C35—C39—O5	−14.1 (3)
C14—C13—C18—C17	0.7 (3)	C36—C35—C39—O5	163.01 (18)
N1—C13—C18—C17	−179.58 (17)	C34—C35—C39—O6	166.52 (18)
C16—C17—C18—C13	0.4 (3)	C36—C35—C39—O6	−16.3 (3)
C20—C17—C18—C13	179.24 (17)	O6—C39—O5—Zn2	28.8 (3)
C16—C15—C19—O2	−10.1 (3)	C35—C39—O5—Zn2	−150.43 (14)
C14—C15—C19—O2	167.10 (17)	O5—C39—O6—Zn1	−6.2 (3)
C16—C15—C19—O1	170.02 (17)	C35—C39—O6—Zn1	173.02 (12)
C14—C15—C19—O1	−12.7 (3)	C36—C37—C40—O7	5.3 (3)
O2—C19—O1—Zn1^iii^	−3.8 (2)	C38—C37—C40—O7	−177.10 (17)
C15—C19—O1—Zn1^iii^	175.98 (13)	C36—C37—C40—O8	−172.62 (17)
O1—C19—O2—Zn2^iii^	3.9 (8)	C38—C37—C40—O8	4.9 (3)
C15—C19—O2—Zn2^iii^	−175.9 (6)	O8—C40—O7—Zn2^iv^	−59.3 (3)
C16—C17—C20—O3	11.8 (3)	C37—C40—O7—Zn2^iv^	122.99 (16)
C18—C17—C20—O3	−166.93 (17)	O7—C40—O8—Zn1^iv^	−8.6 (3)
C16—C17—C20—O4	−167.72 (17)	C37—C40—O8—Zn1^iv^	169.16 (12)
C18—C17—C20—O4	13.5 (2)	C41—N3—C43—O9	−6.4 (4)
O3—C20—O4—Zn1	5.5 (2)	C42—N3—C43—O9	178.5 (2)
C17—C20—O4—Zn1	−174.97 (12)	N3—C43—O9—Zn2	149.88 (19)
C32—N2—C21—C22	−174.83 (19)	C44—N4—C46—O10	−0.8 (3)
C33—N2—C21—C22	−5.6 (3)	C45—N4—C46—O10	174.8 (2)
C32—N2—C21—C26	1.7 (2)	N4—C46—O10—Zn2	−150.09 (18)
C33—N2—C21—C26	170.93 (17)	C47—N5—C49—O11	−1.9 (3)
N2—C21—C22—C23	174.29 (19)	C48—N5—C49—O11	−178.5 (2)

Symmetry codes: (i) −*x*+1, *y*+1/2, −*z*+3/2; (ii) *x*−1, *y*, *z*; (iii) *x*+1, *y*, *z*; (iv) −*x*+1, *y*−1/2, −*z*+3/2.

### Hydrogen-bond geometry (Å, º)

Cg1, Cg3, Cg4 and Cg6 are the centroids of the N1/C1/C6/C7/C12, C1–C6, C7–C12 and C21–C26 rings, respectively.

**Table:**

*D*—H···*A*	*D*—H	H···*A*	*D*···*A*	*D*—H···*A*
O12—H12*O*···O11	0.76	1.93	2.696 (2)	174
C42—H42*B*···O11^v^	0.98	2.52	3.327 (3)	139
C43—H43···O10	0.95	2.44	3.028 (3)	120
C46—H46···O5	0.95	2.39	2.995 (2)	121
C47—H47*B*···O3^iv^	0.98	2.61	3.417 (3)	140
C47—H47*C*···O9^vi^	0.98	2.65	3.608 (3)	165
C2—H2···*Cg*6^vii^	0.95	2.64	3.517 (2)	153
C10—H10···*Cg*3^viii^	0.95	2.64	3.418 (2)	139
C44—H44*B*···*Cg*4^ix^	0.98	2.91	3.596 (2)	128
C45—H45*B*···*Cg*1^ix^	0.98	2.85	3.411 (2)	117

Symmetry codes: (iv) −*x*+1, *y*−1/2, −*z*+3/2; (v) −*x*, *y*+1/2, −*z*+3/2; (vi) −*x*, *y*−1/2, −*z*+3/2; (vii) −*x*+3/2, −*y*+2, *z*−1/2; (viii) *x*+1/2, −*y*+3/2, −*z*+1; (ix) −*x*+3/2, −*y*+2, *z*+1/2.
