# Supplementary figures and images for: Crystal structure of poly[[[μ4-5-(9H-carbazol-9-yl)isophthalato][μ3-5-(9H-carbazol-9-yl)isophthalato]bis­(di­methyl­formamide)(methanol)dizinc] di­methyl­formamide monosolvate]
Source: Acta Crystallogr E Crystallogr Commun. 2015 Jul 31;71(Pt 8):m152–3. doi: 10.1107/S2056989015013961 (PMC4571380; doi:10.1107/S2056989015013961)

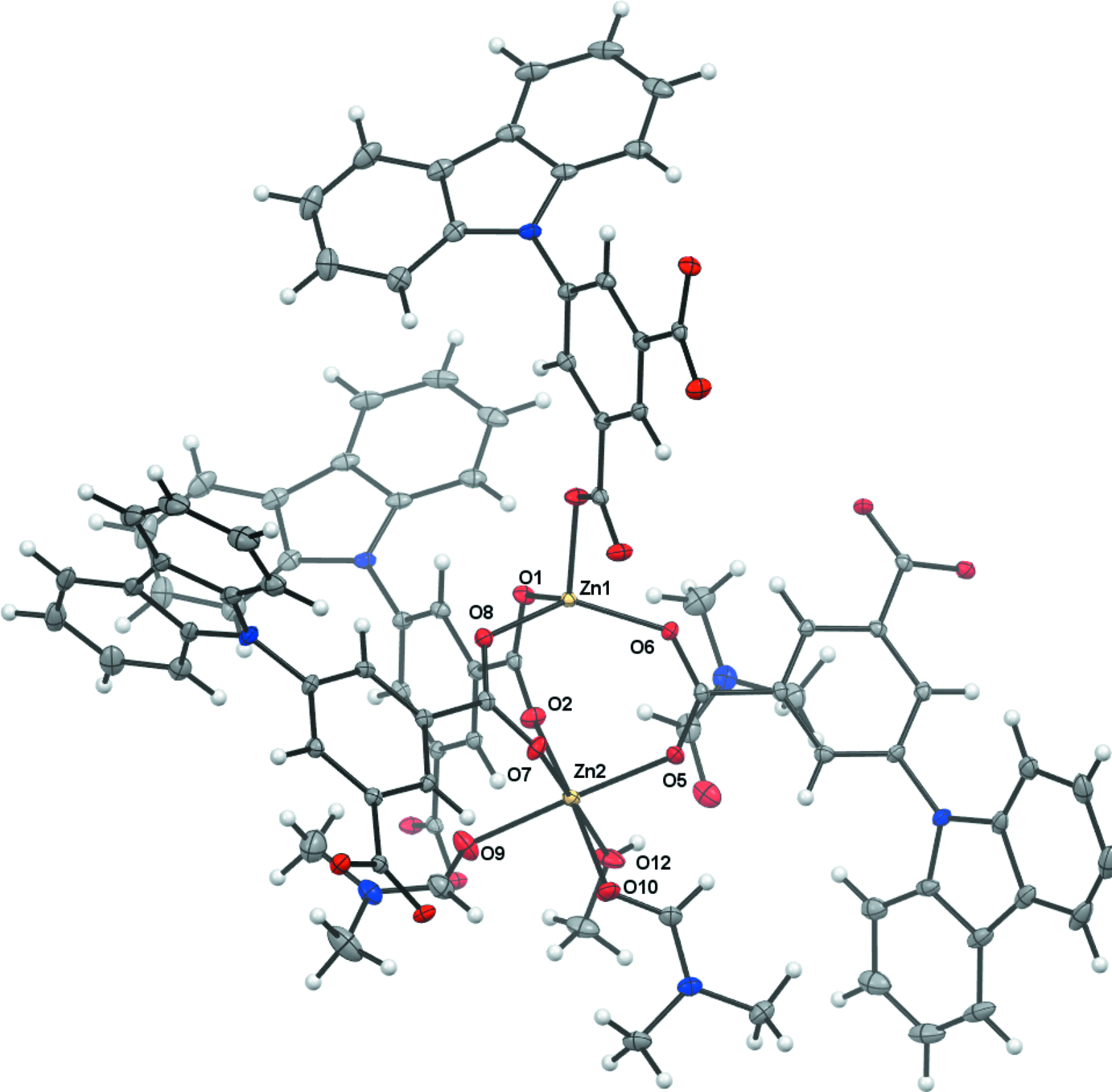

Supplement: Supplementary file 3 [file e-71-0m152-fig1.tif]

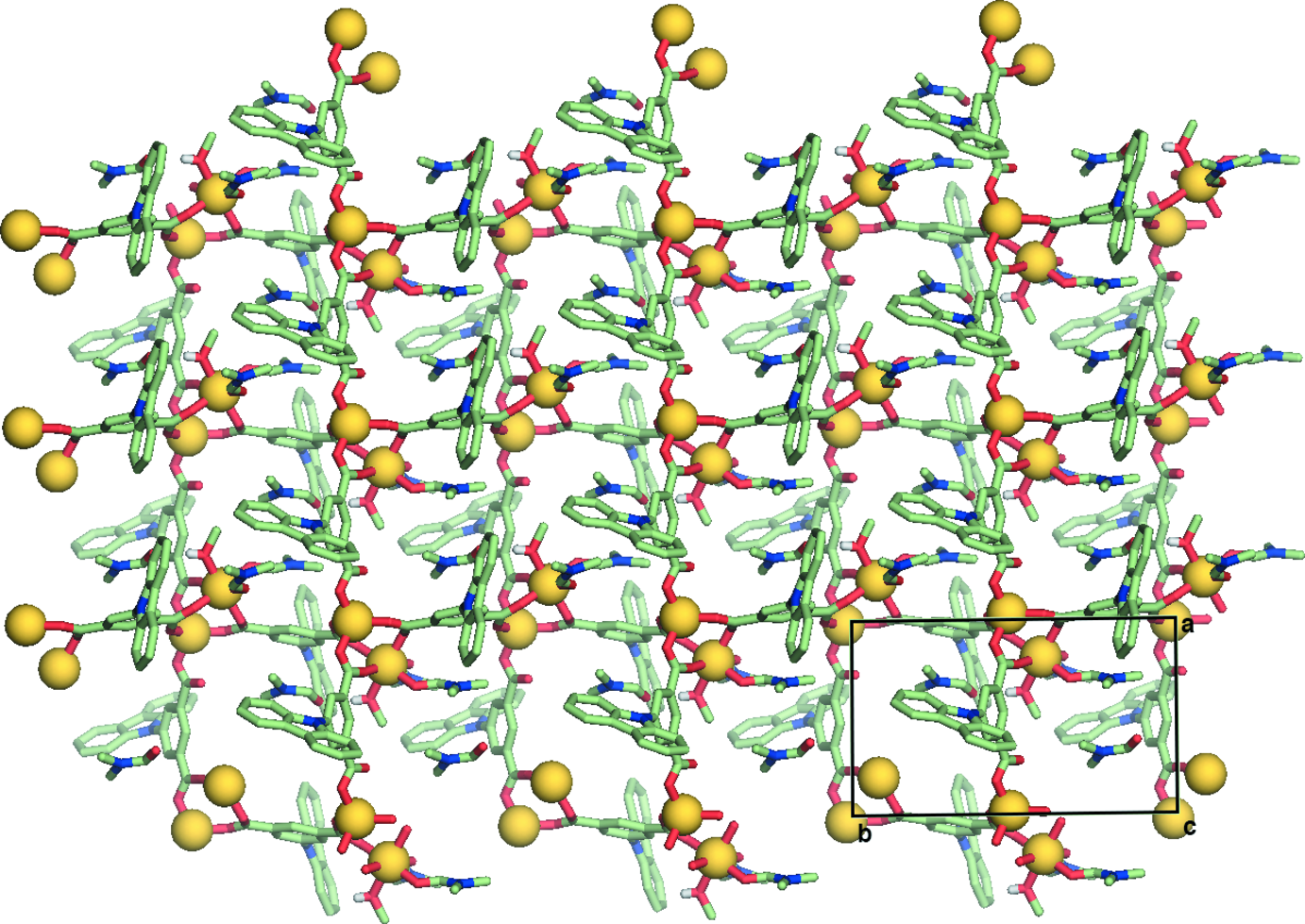

Supplement: Supplementary file 4 [file e-71-0m152-fig2.tif]

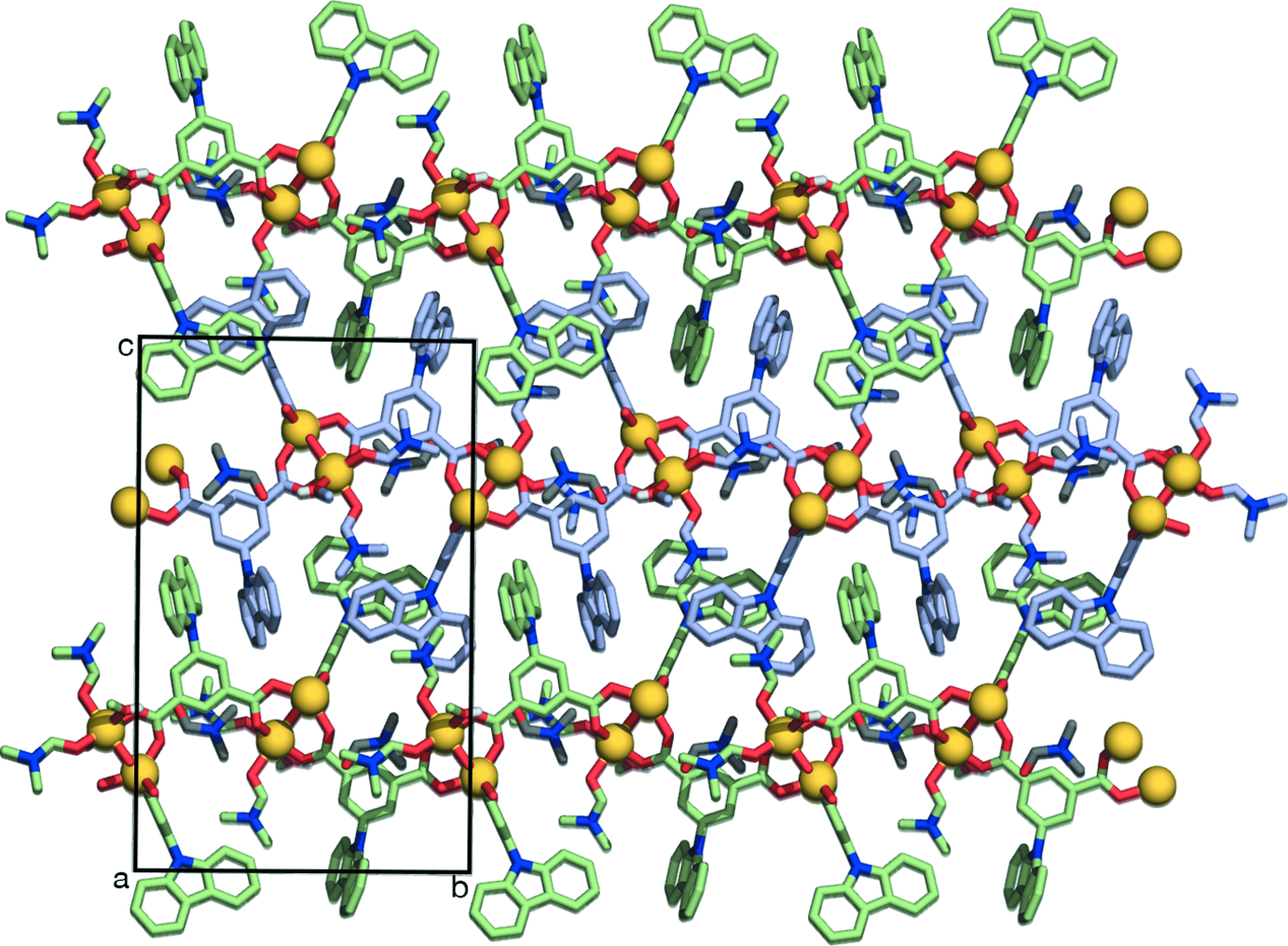

Supplement: Supplementary file 5 [file e-71-0m152-fig3.tif]

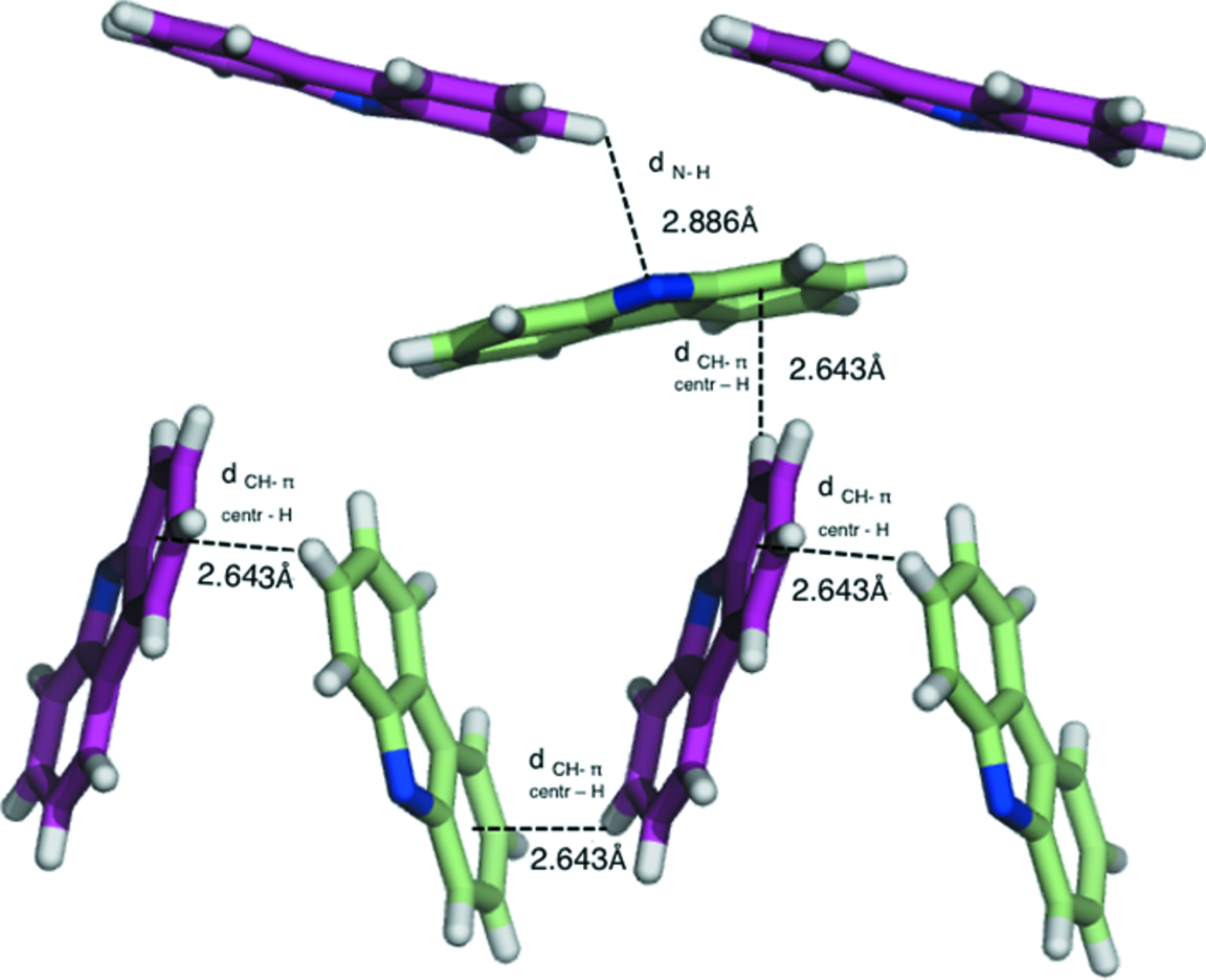

Supplement: Supplementary file 6 [file e-71-0m152-fig4.tif]

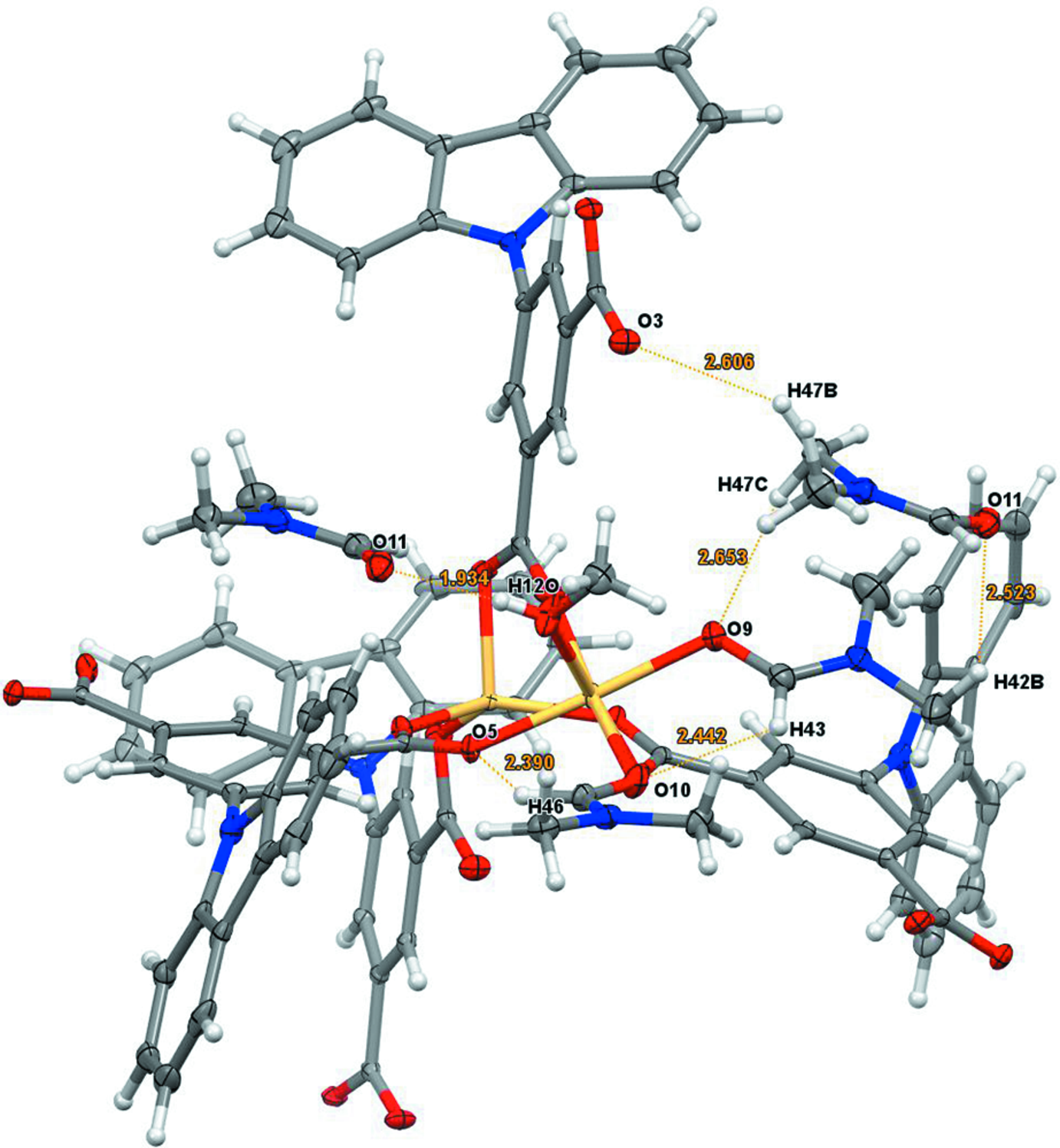

Supplement: Supplementary file 7 [file e-71-0m152-fig5.tif]
